# Insulinoma Masquerading as Rapid Eye Movement Sleep Behavior Disorder: Case Series and Literature Review

**DOI:** 10.1097/MD.0000000000001065

**Published:** 2015-06-26

**Authors:** Keisuke Suzuki, Akiko Kawasaki, Masayuki Miyamoto, Tomoyuki Miyamoto, Takashi Kanbayashi, Masatoshi Sato, Tetsuo Shimizu, Koichi Hirata

**Affiliations:** From the Department of Neurology (KS, AK, KH), Dokkyo Medical University, Tochigi, Japan; School of Nursing (MM), Dokkyo Medical University, Tochigi, Japan; Department of Neurology (TM), Dokkyo Medical University, Koshigaya Hospital, Saitama, Japan; Department of Neuropsychiatry (TK, MS, TS), Akita University School of Medicine, Akita, Japan; and International Institute for Integrative Sleep Medicine (WPI-IIIS) (TK, TS), University of Tsukuba, Tsukuba, Japan.

## Abstract

Insulinoma is a rare endocrine tumor that can cause a wide variety of symptoms, including abnormal nocturnal behavior. We report on 3 patients with insulinoma who presented with abnormal nocturnal behavior and injury during sleep, which simulated rapid eye movement (REM) sleep behavior disorder (RBD). In case 1, the fasting glucose level was 15 mg/dL, and insulin levels were elevated (15 μU/mL). In case 3, when the patient was transferred to the hospital because of a disturbance of consciousness, hypoglycemia (29 mg/dL) was detected. In contrast, in case 2, fasting glucose sampling did not indicate hypoglycemia, but continuous glucose monitoring revealed nocturnal hypoglycemia. The time from initial symptoms to a diagnosis of insulinoma ranged from 7 months to 2 years. All 3 patients had previously received anticonvulsant drugs for suspected epilepsy, but the medications were ineffective. Polysomnography showed no evidence of REM sleep without atonia in any of the 3 patients. No patient remembered any events that occurred during sleep. When a patient manifests abnormal behavior during the night and early morning, glucose monitoring should be performed, especially during the night and early morning. Clinicians should be aware that although insulinomas are rare, they can mimic parasomnias, such as RBD.

## INTRODUCTION

In middle-aged and elderly persons, abnormal nocturnal behavior may result from various diseases and conditions, including nocturnal delirium, sleep-related epilepsy, and metabolic disorders, such as hypoglycemia, as well as parasomnias, such as rapid eye movement (REM) sleep behavior disorder (RBD). Insulinoma is a rare endocrine tumor with an incidence of 1 to 4 in 1,000,000 individuals/y and can occur at any age.^1–3^ Overproduction of insulin due to the neoplastic proliferation of pancreatic islet β cells results in a variety of symptoms of hypoglycemia. Autonomic symptoms include perspiration, tremor, and palpitations, and neuroglycopenic symptoms include personality changes, visual changes, seizures, abnormal behavior, and coma.^1,2^ Additionally, hypoglycemia due to insulinoma can mimic hypersomnia.^4^ In healthy persons, hypoglycemic symptoms are recognized at a plasma glucose level of 60 mg/dL, and brain function impairment occurs at 50 mg/dL during acute insulin-induced hypoglycemia.^2^ In contrast, patients with insulinoma are often free of symptoms despite having critically low glucose levels because of adaptations by the central nervous system to chronic hypoglycemia.^3^ The diagnosis of insulinoma is difficult and is commonly delayed mainly because patients with insulinoma are unaware of their hypoglycemia, and nonspecific symptoms, such as behavioral and psychiatric changes, may be attributed to psychiatric, neurological, or sleep disorders.^1^

Here, we describe 3 patients with insulinoma, including 2 previously reported patients,^5,6^ whose nocturnal symptoms suggest RBD.

## PATIENTS AND METHODS

Between April 2004 and March 2014, 3 insulinoma patients (2 from the Department of Neurology, Dokkyo Medical University and 1 from the Department of Neuropsychiatry, Akita University School of Medicine) whose nocturnal abnormal behaviors suggested RBD were identified. Informed consent was obtained from all patients for being included in the study. Cases 1 and 2 were previously reported.^5,6^ This clinical retrospective observational study did not require ethics committee approval.

## CASE PRESENTATION

### Case 1

A 65-year-old man initially developed stereotypical behaviors (repeatedly opening and closing the bathroom door) and then progressed to more complex behaviors (clapping of hands and walking with sumo-style leg stomps in a circle around the fireplace) that occurred early in the morning. During the abnormal behaviors, he did not communicate with his family members. The patient's abnormal behavior was not related to dream content, and he did not recall any dreams immediately after such episodes. The patient had been treated with haloperidol (0.75–1.0 mg/d) and anticonvulsant drugs, including zonisamide (300 mg/d) and carbamazepine (600 mg/day), for suspected delirium and epilepsy, respectively, at other hospitals at different times; however, neither treatment was effective; instead, his symptoms gradually worsened. Seven months after the initial appearance of his symptoms, the patient was transferred to our hospital for further evaluation.

Brain magnetic resonance images (MRI) showed no abnormality. Electroencephalography (EEG) revealed low-amplitude alpha activity of approximately 10 Hz without epileptic discharges or slow waves. Overnight polysomnography (PSG) did not show REM sleep without atonia. Insulinoma was diagnosed based on a fasting blood glucose level of 15 mg/dL, a high fasting immunoreactive insulin/blood glucose ratio (>0.3), and the presence of a tumor, 12 mm in diameter, that was detected in the pancreas head by abdominal computed tomography (CT). The patient was treated with prednisolone (10 mg/d) orally at bedtime and with glucose (15 g/d). Subsequently, the symptoms of hypoglycemia and the abnormal behaviors disappeared completely.

### Case 2

**FIGURE 1:**
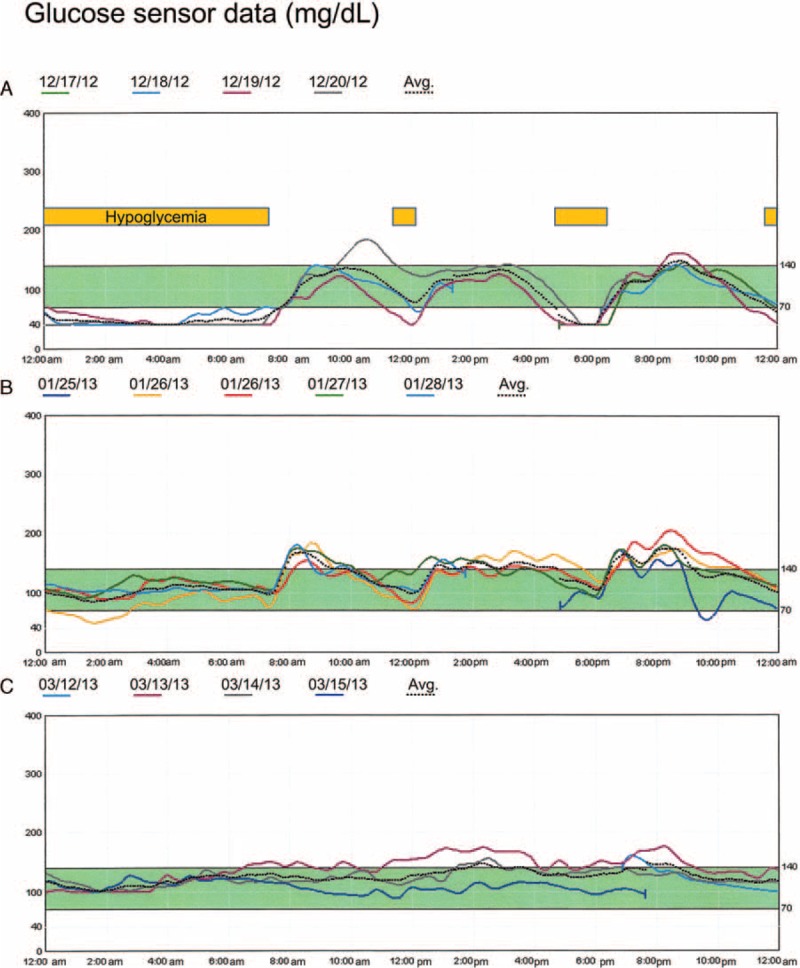
Continuous glucose monitoring in case 2. (A) Nocturnal hypoglycemia was evident before treatment and was significantly improved after diazoxide treatment (B) and tumor resection (C). The yellow bars represent periods of hypoglycemia. The dotted lines represent the average glucose levels during each period.

A 61-year-old woman presented with occasional unusual occurrences around her during the night. The patient lived alone and had no previous medical history. On one occasion, upon waking, she found that the table and chair were both overturned. On a different day, when she woke up, she was lying on the floor, and her face was injured. She did not remember what had happened during the night. At a previous hospital, nocturnal epilepsy was suspected, and the patient was given anticonvulsant drugs. However, her abnormal nocturnal behavior rather worsened. At 63 years of age, the patient was referred to our hospital to be evaluated for parasomnias, including RBD. Laboratory examinations showed a fasting plasma glucose level of 72 mg/dL and an insulin level of 4 μU/mL (normal range: 1.0–17.0). The MRI results were normal. Overnight PSG revealed diffuse 5–6 Hz theta activity on the EEG between 2 episodes of abnormal movements at 0:38 am (after Stage N2 sleep). However, REM sleep without atonia was not detected. A 6-h fast produced hypoglycemia (glucose levels of 40 mg/dL), with insulin levels of 21 μU/mL and C-peptide levels of 3.14 ng/mL. Continuous glucose monitoring (CGM; Medtronic iPro2, Medtronic Japan Co., Ltd, Tokyo, Japan) revealed nocturnal hypoglycemia (Figure 1A). Abdominal dynamic CT revealed an enhanced mass in the pancreatic head. Insulinoma was diagnosed based on these findings. Following treatment with diazoxide (Figure 1B), tumor resection was performed. CGM showed marked improvement (Figure 1C) in nocturnal hypoglycemia, and her abnormal nocturnal behavior completely disappeared.

### Case 3

A 56-year-old woman presented with recurrent episodes of abnormal nocturnal behaviors for 2 years. Once every 2 to 3 months, the patient awakened to find her blanket out of place in her bedroom. One early morning, the patient was found wandering aimlessly, resulting in injuries to her elbow and knee, and was referred to our department to be evaluated for possible sleep disorders. Brain CT and EEG findings at a previous hospital were unremarkable. Overnight PSG did not show REM sleep without atonia or complex motor behaviors during REM sleep. However, RBD was suspected due to the episodes of injury that had occurred during the night. Clonazepam was started at a dosage of 0.5 mg at bedtime; this dosage was increased to 1.0 mg, but the frequency of abnormal nocturnal behavior did not change. Thereafter, episodes of groaning and twisting her trunk and bilateral arms suggested epilepsy, and treatment with anticonvulsant drugs was initiated. However, neither carbamazepine (200 mg/d) nor topiramate (200 mg/d) was effective. Moreover, continuous EEG monitoring did not detect any epileptic discharges. One month later, the patient was admitted to the hospital because of deep coma. Laboratory examinations revealed hypoglycemia (29 mg/dL). An intravenous infusion of glucose rapidly reversed hypoglycemia and increased her level of consciousness. A tumor approximately 10 mm in diameter was detected in the pancreas head by abdominal enhanced CT and MRI. These results, together with fasting test results, led to a diagnosis of insulinoma, and surgical resection of tumor was performed. The patient's condition had been stable, without recurrence of abnormal behaviors, over a 2-month follow-up period.

## RESULTS

Table 1 shows the characteristics of the 3 patients. All 3 patients had previously received anticonvulsant drugs for suspected epilepsy, but the medications were ineffective. PSG showed no evidence of REM sleep without atonia in any of the 3 patients. No patient remembered any events that occurred during sleep. However, RBD was suspected based on nocturnal abnormal behaviors and several episodes of injury during the night. The period from the initial appearance of symptoms to a diagnosis of insulinoma ranged from 7 months to 2 years.

**TABLE 1:**
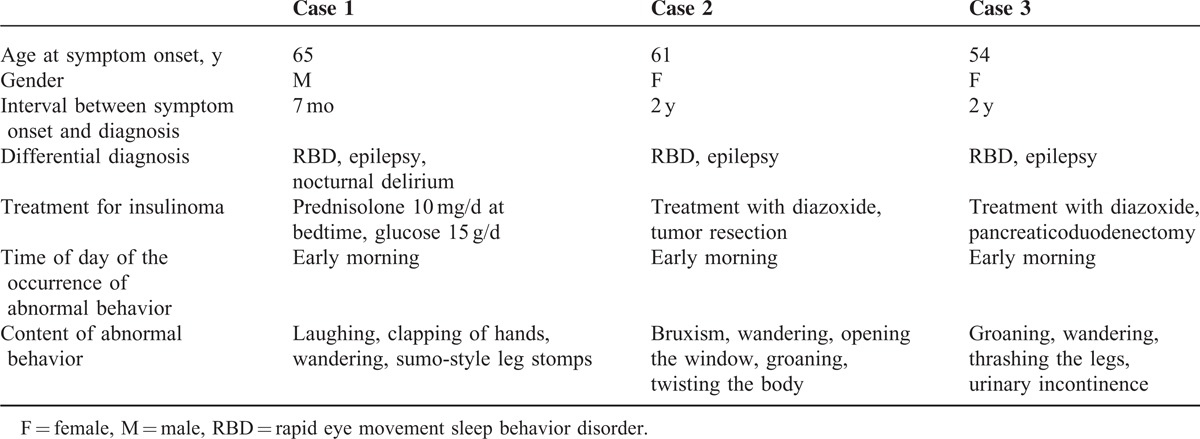
Clinical Characteristics of 3 Patients With Insulinoma

## DISCUSSION

We have described 3 patients with insulinoma whose abnormal nocturnal behaviors suggested RBD. Other disorders in the differential diagnosis of insulinoma included night delirium and epilepsy. Table 2 shows disorders in the differential diagnosis of abnormal nocturnal behavior. Epilepsy was suspected, and anticonvulsant drugs were administered to all of the patients without clinical efficacy. Graves et al^7^ described a 44-year-old woman with multiple insulinomas who presented with adult-onset refractory seizures; the time from symptom onset to diagnosis was 4.5 years for this patient. In a previous study that examined 59 histologically confirmed cases of insulinoma, the interval from symptom onset to diagnosis ranged from 1 month to 30 years (median, 2 years).^8^ In contrast, in our series the period from the initial appearance of symptoms to a diagnosis of insulinoma ranged from 7 months to 2 years. Therefore, increasing the clinical suspicion of insulinoma in patients presenting with abnormal nocturnal behaviors is essential to prevent diagnostic delay and resulting neurological sequelae, including cognitive impairment. Wandering and bizarre and complex behaviors are common in our patients; however, RBD has not been widely recognized as an important disorder in the differential diagnosis of insulinoma.

**TABLE 2:**
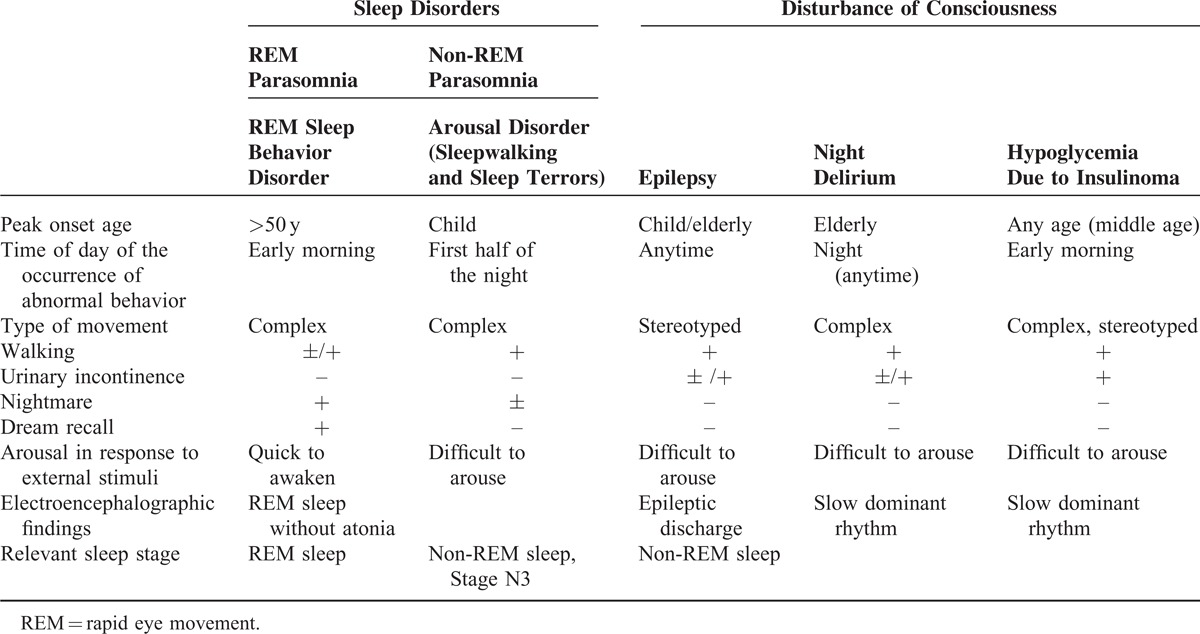
Differential Diagnosis of Abnormal Nocturnal Behavior: Sleep Disorders Versus Disturbance of Consciousness

RBD is characterized by dream-enacting behavior and a loss of normal muscle atonia during REM sleep, often resulting in injury to the patient or the patient's bed partner. RBD patients show electromyographic abnormalities during REM sleep; they exhibit elevated muscle tone during REM sleep, a phenomenon called REM sleep without atonia.^9^ PSG findings showed no evidence of REM sleep without atonia in our 3 patients; however, this lack of evidence cannot exclude RBD completely, given the night-to-night variability of PSG. The *International Classification of Sleep Disorders*^9^ states that for patients who had typical episodes of dream-enacting behavior and showed complex motor behaviors during PSG but did not show sufficient REM sleep without atonia, RBD may be provisionally diagnosed based on clinical judgment. However, patients with RBD typically awaken quickly, become rapidly alert, and can recall the contents of their dreams upon awakening, whereas patients showing abnormal nocturnal behaviors related to other disorders, such as night delirium, epilepsy, and hypoglycemia due to insulinoma, cannot awaken quickly or report the contents of dreams.^9^ The abnormal behaviors of our 3 patients were not related to any dream content during sleep, and none of the patients awakened rapidly from sleep, confirming that a dream can be recalled is thought to be important for distinguishing RBD from a disturbance of consciousness related to a metabolic disorder or epilepsy. It is difficult to differentiate insulinoma from non-REM parasomnia, epilepsy, and night delirium based on clinical histories alone.

**FIGURE 2:**
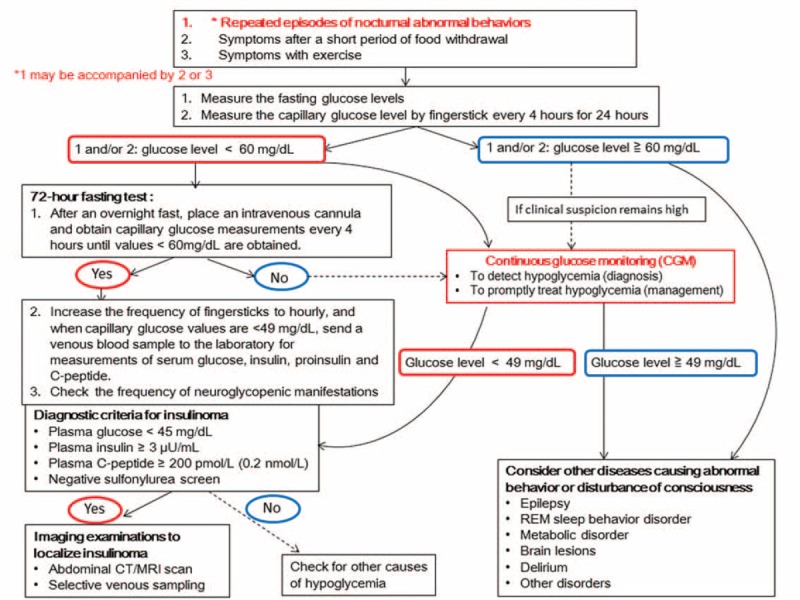
Flow chart illustrating the differential diagnosis of repeated episodes of abnormal nocturnal behavior. For patients with insulinoma, detecting hypoglycemia via a fasting test and/or CGM is essential before performing imaging studies (CT/MRI or selective venous sampling). CGM = continuous glucose monitoring, CT = computed tomography, MRI = magnetic resonance images.

Hypoglycemia is classified based on an insulin-mediated or a noninsulin-mediated condition. The most common cause of hyperinsulinemic hypoglycemia is reported to be insulinoma.^10^ In case 1, the fasting glucose level was 15 mg/dL, and the insulin level was elevated (15 μU/mL); in case 3, hypoglycemia (29 mg/dL) was detected when the patient was admitted to the hospital because of a disturbance of consciousness. In contrast, in case 2, fasting glucose sampling did not show hypoglycemia, but CGM revealed nocturnal hypoglycemia, which suggests the importance of the use of CGM. We provide a flowchart, constructed based on the work of Masharani, illustrating the differential diagnosis of insulinoma (Figure 2).^11^ Several studies have demonstrated the clinical utility of CGM in the diagnosis of insulinoma and the management of glucose levels in unresectable insulinomas.^1^ CGM is also useful for localizing a tumor and providing evidence of the complete removal of a tumor.^12^

In healthy individuals, despite the prolonged fasting condition that is experienced during sleep, glucose levels remain stable during the night, including during REM sleep (marginal variation). However, the potential relationship between sleep stages (including REM sleep) and glucose levels in insulinoma patients has not been studied. Sangiah et al^13^ observed a sequential reduction in REM sleep time and an increase in non-REM sleep time following the administration of a nonconvulsive dose of insulin in rats. The authors suggested that the insulin directly affected the sleep–wake cycle by possibly acting on neurotransmitters, such as serotonin, catecholamines, and acetylcholine, which modulate the sleep–wake cycle in rats.^14^ One study evaluated the insulin/glucose ratio during sleep in 4 patients with insulinoma. The insulin/glucose ratio was highest, and the glucose levels were lowest between 3 am and 6 am. Although PSG was not performed, the authors suggest that the abnormal secretion of insulin and hypoglycemia that occurs in insulinoma patients during night is due to the incapacity to react normally to the prolonged fasting condition or to the sleep itself.^15^ In case 2, during PSG, we observed episodes of abnormal movements that were likely due to hypoglycemia, and these abnormal movements occurred after Stage N2. However, the glucose levels were not monitored simultaneously. Little evidence exists supporting a relationship between sleep stages (including REM sleep), glucose levels, and neuroglycopenic symptoms in insulinoma patients. However, we believe that hypoglycemia in insulinoma patients is unlikely to produce RBD arising from REM sleep. Rather, we hypothesize that the neuroglycopenic symptoms observed in insulinoma patients during sleep, which directly result from glucose deprivation within the central nervous system, mimic RBD for several reasons. First, RBD likely occurs during the early morning when the REM sleep time is the longest. For insulinoma patients, neuroglycopenic symptoms also tend to occur during the early morning following prolonged fasting. There are similarities in the complex, often violent, abnormal movements and behaviors observed in patients with RBD and insulinoma. These behaviors contrast the stereotypical, repetitive movements observed in epilepsy patients.

The limitations of this study include the retrospective observational design and small number of participants. Additionally, the simultaneous monitoring of sleep stage (using PSG) and glucose level could not be performed in this study. However, it may be ethically immoral to perform simultaneous monitoring in insulinoma patients without continuous glucose administration following the diagnosis of insulinoma.

In conclusion, because insulinoma can present with various symptoms, including abnormal nocturnal behavior, clinicians should be aware that although insulinomas are rare, they can mimic parasomnias, such as RBD.
